# The Effect of Aging Process Conditions on the Thermal Properties of Poly(Dimethylsiloxane)-Based Silicone Rubber

**DOI:** 10.3390/ma17225608

**Published:** 2024-11-16

**Authors:** Anna Morawska-Chochół, Magdalena Szumera, Andrzej Młyniec, Kinga Pielichowska

**Affiliations:** 1 Department of Biomaterials and Composites, Faculty of Materials Science and Ceramics, AGH University of Krakow, al. Mickiewicza 30, 30-059 Krakow, Poland; morawska@agh.edu.pl; 2 Department of Ceramics and Refractories, Faculty of Materials Science and Ceramics, AGH University of Krakow, al. Mickiewicza 30, 30-059 Krakow, Poland; mszumera@agh.edu.pl; 3 Space Technology Centre, AGH University of Krakow, al. Mickiewicza 30, 30-059 Krakow, Poland; mlyniec@agh.edu.pl

**Keywords:** PDMS, silicone rubbers, aging, thermal analysis, molecular structure

## Abstract

Silicone rubbers based on poly(dimethylsiloxane) (PDMS) are crosslinked elastomers commonly used in various branches of industry, especially as packing materials in elements for high-temperature service. In addition to high temperatures, mechanical loading may influence their structure during their work, and, as a consequence, their thermal properties may change. This study’s findings on the degradation mechanism under aging conditions are not just necessary, but also crucial for their satisfactory application. The aim of the study was a detailed and comprehensive evaluation of the aging processes of commercial ELASTOSIL^®^ LR 3842/50 A/B, considering structural changes based on thermal analysis accompanied by mass spectroscopy, X-ray analysis, and infrared spectroscopy. The aging process was carried out at 125 °C and 175 °C, without and with 11 kg of loading. The obtained results showed that the aging of PDMS increased their thermal stability. It was the most visible for PDMS aging at 175 °C under load. It was attributed to secondary crosslinking and the post-curing process. Observed changes in polymer structure did not indicate its degradation. This is a significant finding, especially considering that a temperature of 175 °C is close to the critical temperature given by the producer (180 °C), above which the use of stabilizing agents is recommended.

## 1. Introduction

Silicone rubbers based on poly(dimethylsiloxane) (PDMS) are common materials used in a variety of branches of industry, mainly due to their high thermal stability, high dynamic flexibility, high oxidative stability and lubricity, as well as low glass transition temperature [1,2,3]. However, their properties and, as a consequence, their application, are closely related to their molecular structure. Silicones, as typical elastomers, are characterized by low glass temperature (T_g_), whose value is usually contained in the range of −120 °C to −100 °C. Above T_g_, segmental movements of the polymer chain are possible, and they consist of coordinated changing of monomeric units. Therefore, relocating polymer chains to each other without breaking bonds is possible under the influence of loading. After loading is eliminated, the chains return to their original form. This behavior is possible because of the strong bonding between polymer chains and the effect of partial crosslinking during the vulcanization process with catalysts.

The main criteria influencing the thermal properties include molecular weight, predominantly linear or branching structure, crosslinking degree, flexibility of the –[Si–O]_x_– chain segments, and cyclic siloxanes [4,5]. Thermal stability increases significantly with increasing molecular weight and crosslinking degree and usually exceeds 300 °C. However, it is higher for cyclic siloxanes with a lower molecular weight than for their linear equivalents with a higher molecular weight [6]. The high thermal stability of polysiloxanes, including poly(dimethylsiloxanes), is a consequence of the high energy of the Si-O bond (461 (kJ/mol), which considerably exceeds the energy of bonds characteristic of typical organic polymers, such as the C-C bond (304 kJ/mol) or C-O bond (358 kJ/mol) [7].

The typical mechanism of thermal degradation of PDMS occurs through two competing reactions, molecular and radical. The molecular response consists of Si-O bond scission and leads to the formation of cyclic oligomers, whereas the radical reaction progresses through homolytic Si-CH_3_ bond scission with the release of methane through hydrogen abstraction, and this process dominates at high temperatures [8,9]. The coupling of radicals during the reaction leads to the crosslinking of the macroradicals and decreases the PDMS chain’s flexibility. Splitting of cyclic oligomers is hindered during the further stage [6]. During the reaction, along with increasing crosslinking, the thermal stability of PDMS is also observed [10].

As crosslinked elastomers and because of their high thermal stability, PDMSs are considered for application as packing materials in elements for high-temperature service. In addition to high temperatures, mechanical loading may influence their structure during work; consequently, their thermal properties may change. Knowledge of their degradation mechanism under the influence of aging conditions is necessary to apply these materials in packaging services successfully. It must be considered that long-term high-temperature action on polymeric materials, even in the safe working range, may partially cause degradation [11,12].

In siloxanes, in the presence of moisture, the reversible reaction of the Si-O bond breaks can be observed, leading to the formation of Si-OH groups (silanols). As a result of silanol formation, the loss of hydrophobicity and adhesive property was observed due to the migration of hydrophilic Si-OH groups onto the exposed surface. In extreme cases where moisture exposure is prolonged, surface cracking also occurs [13,14]. Exposure to heat can also cause degradation of the siloxane network, which results in hardening of the bulk polymer. At temperatures above 180 °C, a thermal decomposition occurs in which the siloxane chain is shortened, and a cyclic compound is formed. The formation of low-molecular-weight cyclic siloxanes leads to a loss of elasticity due to the cyclic compounds’ volatility and the polymer chain’s destruction [15].

Moreover, Vallés et al. [16] investigated the influence of the thermal weathering of polydimethylsiloxanes on molecular weight distributions: they observed a loss of average molecular weight and a broadening of molecular weight distributions. Patel and co-workers [17] reported on the thermal degradation of condensation-curing polysiloxane rubbers exposed to sealed and open-air aging regimes. They found that aging conditions cause significant changes in the non-network phase and the crosslinked polymer structure. They observed that accelerated thermal aging under open-to-air conditions due to the loss of low-molecular-weight volatile species, while suppressing volatile emission, as in sealed aging, promotes cure reversion. Moreover, they revealed siloxane rubbers aged in a closed system softened with time, whereas samples aged open to air did not show this behavior [18]. Visser et al. [19] demonstrated that cyclic stress accelerates siloxane degradation. Mechanical forces can also enhance or alter the rate of degradation. Stress can result in chain scission and the production of highly reactive chain fragments, which initiate various degradation reactions. Stress relaxation studies indicated that crosslinking and chain scission occurred in unfilled PDMS elastomers at different temperatures [20].

Silicone materials such as PDMS are widely used in countless industries, ranging from advanced sealing and vibration damping components, to micro-fabricated components in biomedical engineering such as lab-on-a-chip devices, to common kitchen equipment, due to their temperature, chemical and mechanical resistance, and ease of forming complex shapes. In many applications, their high resistance to the thermal stresses that accompany mechanical loading is required. Our research fills a research gap in knowledge in this area. Despite many scientific papers related to the study of aging processes and the degradation of polysiloxanes, some discrepancies still need to be made visible. These result from differences in their molecular structure and the additives used. It translates into difficulties in determining the stability of PDMS resins under conditions of long-term operation at elevated temperatures and under load.

The study aimed to evaluate the aging processes of commercial ELASTOSIL^®^ LR 3842/50 A/B (Shore 50), considering structural changes based on thermal analysis accompanied by mass spectroscopy, X-ray analysis, and infrared spectroscopy. The aging process was conducted at 125 °C and 175 °C; the latter is close to the temperature of safe operation of the material without stabilizing agents, specified by the producer (180 °C). Aging was carried out without and with 11 kg of loading.

## 2. Materials and Methods

Silicone rubber was produced by Wacker Chemie AG (Munchen, Germany) with the trademark ELASTOSIL^®^ LR 3842/50 A/B (Shore 50).

The producer provides the following composition of the material: ELASTOSIL^®^ LR 3842/50 A/B —silicon rubber low-soaked in oil, obtained from two components, component A—ELASTOSIL^®^ LR 3842/50 A with chemical composition: poly(dimethylsiloxane) with vinyl groups + auxiliaries; component B—ELASTOSIL^®^ LR 3842/50 B with chemical composition: poly(dimethylsiloxane) with functional groups + auxiliaries.

The samples with a cylindrical shape were aged in two temperatures (125 and 175 °C) without loading and after 11 kg loading.

Sample designations:

PDMS_50—fresh PDMS

PDMS_50_0_125—PDMS without loading after aging at 125 °C

PDMS_50_11_125—PDMS after 11 kg loading at 125 °C

PDMS_50_0_175—PDMS without loading after aging at 175 °C

PDMS_50_11_175—PDMS after 11 kg loading at 175 °C

The use of the simplest loading with weights was due to the need for long-term conditioning of the samples in the same ovens that are used for product validation. This was due to the need to reflect the environmental conditions in the engine compartment, which are simulated by standardized SAE/USCAR tests. The tests were performed on dozens of samples simultaneously, and lasted 500 h, which would not have been possible using other testing methods. In our opinion, the use of static loading does not adversely affect the result of the work carried out. The chosen temperatures for testing the long-term properties of PDMS are a direct result of the test conditions defined by the SAE/USCAR-2 rev.5 standard, according to which the product validation tests were conducted for the products in which the test materials were used. The USCAR standard defines aging tests for sealing materials at two temperatures, depending on the seal’s location within the vehicle. Tests last 500 h at a temperature of 125 °C, and for seals located closer to the source of heat, at 175 °C.

The defined load value was chosen to match the dimensions of the specimen so that the initial deformation does not exceed 35%, above which we observed in validation tests of finished sealing and damping components significant deformation of the component leading to a loss of its functionality.

Structural studies by X-ray diffraction were carried out on the Philips X’Pert Pro X-ray diffractometer (Panalytical, Malvern, Worcestershire, UK), equipped with a two-wheeled powder diffractometer with a goniometer diameter of 240 mm.

The FTIR spectra of the tested materials were made using the ATR technique on Bio-Rad (Hercules, CA, USA) equipment, FTS 3000 Excalibur Series, Miracle ATR, on a diamond crystal with zinc selenide (ZnSe) optics from Piketech.

Microscopic observations and element analysis were made on the Nova 200 NanoSEM scanning electron microscope manufactured by FEI Company with an attachment for elemental composition analysis (EDAX). Tests of the surface of the samples were carried out without carbon spraying to obtain a thorough analysis of the composition, which, however, takes place at the expense of the quality of the images. Cross-sections of the samples were tested after carbon spraying.

TG (thermogravimetric analysis) and DSC (differential scanning calorimetry) measurements were performed on the NETZSCH STA 449 F3 Jupiter^®^ analyzer (Selb, Germany). Samples with mass ca. 6 mg were heated in pierced aluminum pans in an air atmosphere at a heating rate of 10 °C/min to 600 °C. The device was prepared for the tests by calibrating it both in terms of sensitivity calibration and temperature. For this purpose, specialist reference materials supplied by Netzsch were used, guaranteeing the achievement of results at a high scientific level. Thermogravimetric analysis coupled with mass spectroscopy (TG-MS) was performed with a TA Instruments 2960 SDT thermal analyzer with a heating rate of 20 °C/min (sample mass ca. 5 mg, airflow 100 cm^3^/min) and a Balzers Thermo-Star quadrupole mass spectrometer (New Castle, DE, USA). The ionizing voltage of the cross-beam electron impact ionization source was 70 eV. The TG analyzer and MS were coupled to enable the passage of evolved products from the furnace to the gas cell over a short-heated path to minimize secondary reaction or condensation. DSC measurements were performed using a DSC 1 (Mettler Toledo, Greifensee, Switzerland) calibrated with indium and cooled with liquid nitrogen. Samples of approximately 5 mg were placed in standard aluminum pans. Measurements were conducted in the temperature range −120 to 70 °C with a 10 °C/min rate under argon atmosphere.

## 3. Results and Discussion

**Figure 1:**
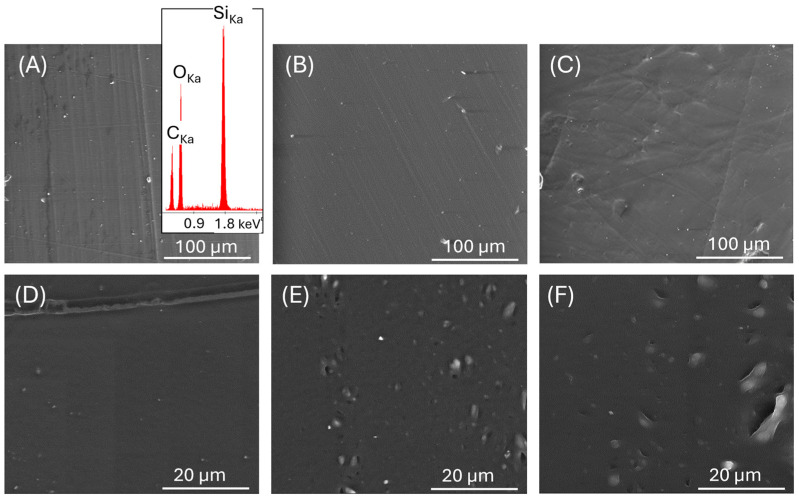
SEM image of surface: PDMS_50 with EDS analysis (**A**); PDMS_50_0_175 (**B**); PDMS_50_11_175 (**C**); SEM images of samples cross-section: PDMA_50 (**D**); PDMS_50_0_175 (**E**); PDMS_50_11_175 (**F**).

The influence of aging on PDMS properties can be considered at different levels: microstructural, structural, and molecular. Changes in the microstructure of samples were estimated by microscopic analysis of their surface and cross-sections. SEM observations and EDS analysis were performed for the surface of the fresh PDMS and after aging and loading (Figure 1). EDS analysis confirmed the presence of silicone only (Figure 1A). The surfaces of PDMS_50 and PDMS_0_175 were smooth without any defects related to high-temperature acting (Figure 1A,B). After loading at 175 °C, the surface morphology was quite different; the unevenness and corrugation were visible (Figure 1C). It indicates irreversible deformation of the sample surface under 11 kg loading and high temperature. The microstructures of the sample cross-section were also tested (Figure 1D–F). For the initial sample, the cross-section was very smooth; however, after aging at 175 °C, the pores became more pronounced. Moreover, the pores were even larger after loading the PDMS at 175 °C. The creation of pores can be related to gradually removing the silicone lubricant introduced at the production stage. As shown in the thermal analysis described below, decomposition or evaporation of low-molecular silicone lubricant occurred at lower temperatures than PDMS thermal degradation.

**Figure 2:**
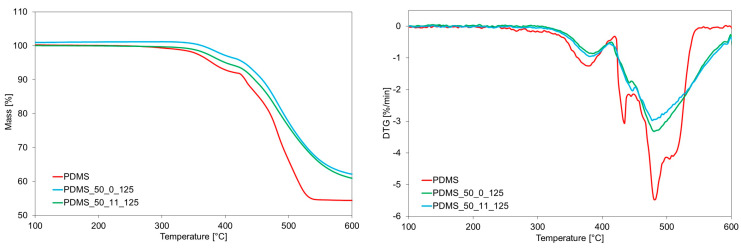

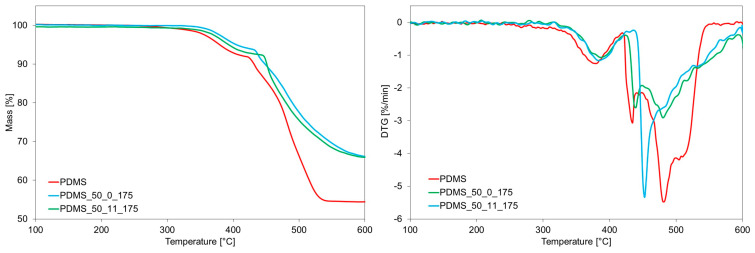
TG and DTG curves for investigated samples before and after aging at 125 °C and 175 °C with and without loading.

**Figure 3:**
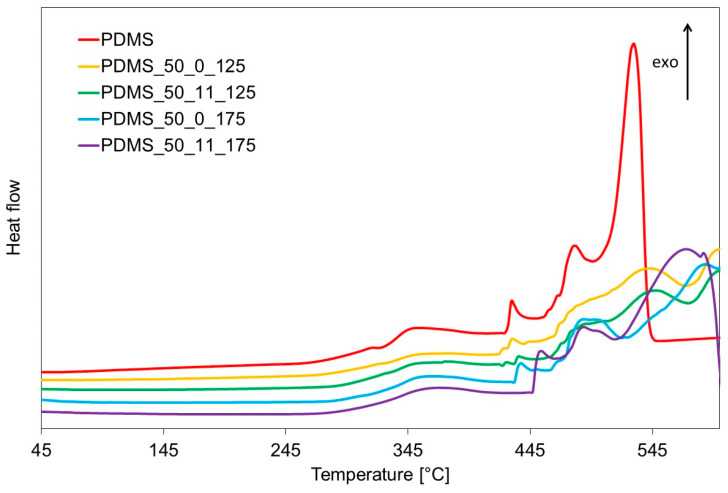
DSC curves for fresh sample and after aging at 125 °C and 175 °C (with and without loading).

Not only can the aging process affect the microstructure, but above all, it can impact the structure and molecular organization. Therefore, thermal analysis was performed. Results of thermogravimetric analysis of virgin PDMS and PDMS after aging at 125 °C and 175 °C are presented in Figure 2 and Table 1. It can be seen that the degradation starts at a temperature higher than 300 °C. Furthermore, as can be seen from Table 1, aging and loading significantly influence the thermal stability of the investigated samples—one percent of mass change is observed for virgin PDMS at the temperature of 306 °C, while for aged samples, it is in the range of 347–362 °C. From TG curves, decomposition proceeds in two significant steps. However, from the DTG curve, it can be seen that in the second step, two or three partially overlapped processes can be observed, and the char residue is large (up to 65.9%). It is also confirmed in the DSC curves, as seen in Figure 3. It should be noted that the amount of char residue increases after aging and with the temperature of aging—for samples aged at higher temperatures, the amount of char residue is higher.

The lower thermal stability of virgin samples can be attributed to the partial decomposition or evaporation of the silicone’s low-molecular lubricant or lower crosslinking density. The lubricant was incorporated into the sample during the production process at a weight of 2%, as described in the product specification. Moreover, the main chain structure has comonomer units with free vinyl groups that enable free radical polymerization and crosslinking of the polymer. In this context, the improvement of thermal stability during the aging process can also be attributed to the change in PDMS structure through crosslinking because of the post-curing effect.

During the thermal aging of PDMS in closed systems, polymeric silanols are formed due to hydrolysis in the presence of some water. Zeldin et al. [21] have proposed a siloxane hydrolysis mechanism that leads to the build-up of low-molecular-weight cyclic fragments in the rubber network. PDMS hydrolysis can lead to loss of crosslink density if the cyclic fragments contain a long segment of the polymer backbone.

Thermo-oxidative degradation of pure PDMS using an evolved gas analysis coupled with thermogravimetric analysis (TG) was studied by Grassie et al. [22], who demonstrated that PDMS degrades via a depolymerization reaction to yield cyclic oligomeric siloxanes. The depolymerization proceeds from both free chain ends and results from intramolecular backbiting reactions of continuous chain segments. Furthermore, with decreasing inter-crosslink chain length, larger cyclic siloxane species (>D5) become more abundant degradation products, and there is a relationship between the interchain molar mass, the degree of crosslinking, and the degradation thermal stability concerning the mechanisms [23].

According to Lewicki and colleagues [23], the first degradation step (primary degradation in the range of 350–450 °C) can be attributed to a series of terminal and internal intramolecular chain backbiting reactions and cyclic siloxanes formation. The second step in the range of 450–600 °C is generally understood to involve radically induced crosslinking and high-temperature –CH_3_ abstraction reactions to form branched species, dicyclics, and small molecules such as CH_4_ and H_2_. During secondary degradation, a few different processes can take place, as observed in the presented results. In addition, it seems that loading during thermal aging has a more significant influence at higher temperatures: for samples aged at 125 °C, the DTA and DSC profiles for secondary degradation look similar, while for samples aged at 175 °C, they differ quite significantly. Additionally, lower thermal stability is observed in samples aged under loading conditions—under loading, the diffusion processes and chain segment movement necessary for backbiting and crosslinking processes are more hindered than in samples without loading. 

**Figure 4:**
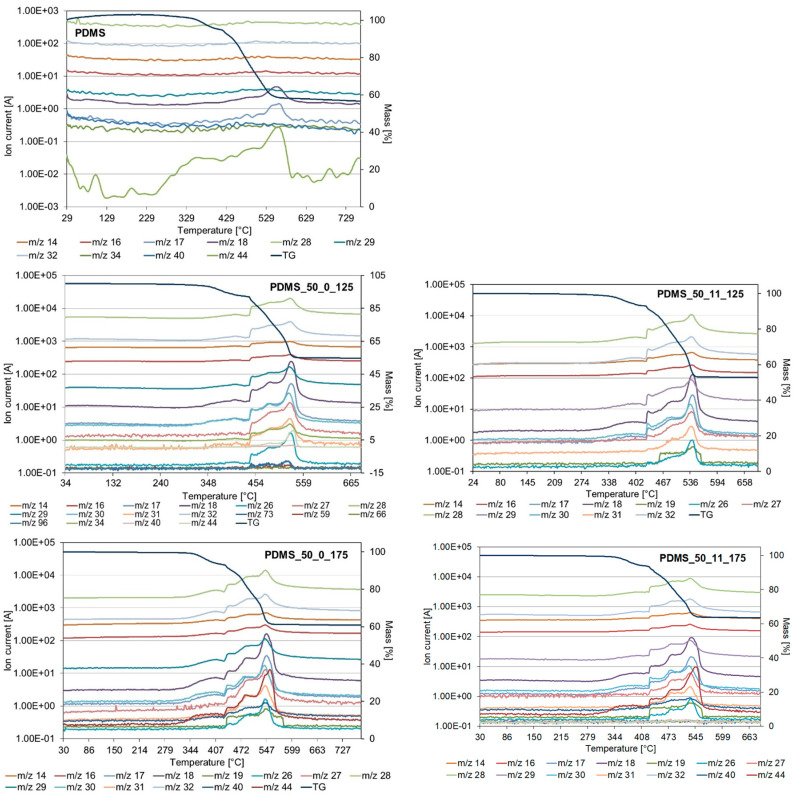
TG-MS results for fresh sample and after aging at 125 °C and 175 °C (with and without loading).

Thermoanalytical investigations using the TG-MS method have been performed to gain deeper insights into the thermal degradation processes. As can be seen from Figure 4, there are some differences in the shape and intensity of fragmentation ions released by PDMS and PDMS after aging. It should also be noted that the pattern looks quite similar for all PDMS samples after aging. It suggests that PDMS aging leads to processes inside samples that change the thermal degradation mechanism. Grassie et al. [22] reported that the thermal decomposition of linear chain PDMS undergoes complete depolymerization by the evolution of a spectrum of cyclic oligomers ranging from D3 to D12 units. The PDMS degradation products were mainly cyclic oligomers like D3 (trimer 46%) and D4 (tetramer 20%), as well as lower amounts of D5 (9%), D6 (11%), D7(8%), and D8 (3%). Higher oligomers D9-D11 were detected in smaller amounts in decreasing proportions [24]. According to Venkatachalam and Hourlier [25], the first step of PDMS thermal degradation up to 400 °C under inert conditions leads to the water (*m*/*z* 18), and silicon-based species like (CH_3_)_3_-Si-OH (*m*/*z* 75, 45, 47, 29, 43) and (CH_3_)_3_-SiH (*m*/*z* 59, 73, 43, 58). In the presence of water, PDMS undergoes hydrolysis with release species of (CH_3_)_3_Si-O- and (CH_3_)_3_Si-OH (*m*/*z* 75, 45). The presence of (CH_3_)_3_SiH (*m*/*z* 59, 73) and (CH_3_)_2_SiH_2_ (*m*/*z* 59, 44, 28) is connected to the release of the end sites. During the second degradation step in the temperature range 400–470 °C, the removal of hydrocarbon species such as methane CH_4_ (*m*/*z* 16, 15, 14, 12) and ethylene C_2_H_4_ (*m*/*z* 28, 27, 26, 25), derived from the vinyl terminal sites of the base PDMS, and the decomposition of Si-C_2_H_3_ were detected. At a temperature ranging from 480 °C to 600 °C, silicon-based oligomers (linear molecules (CH_3_)_3_–Si–O–Si–(CH_3_)_3_ (*m*/*z* 147, 73, 66), cyclic molecules D3 or D5 ([(CH_3_)_2_–SiO]_3_, or [(CH_3_)_2_–SiO]_5_ with *m*/*z* 207, 96, 191, 133 or *m*/*z* 73, 355, 267, 45, 59, respectively) were found. At this step, the elimination of water and hydrogen was also observed [26,27,28].

**Figure 5:**
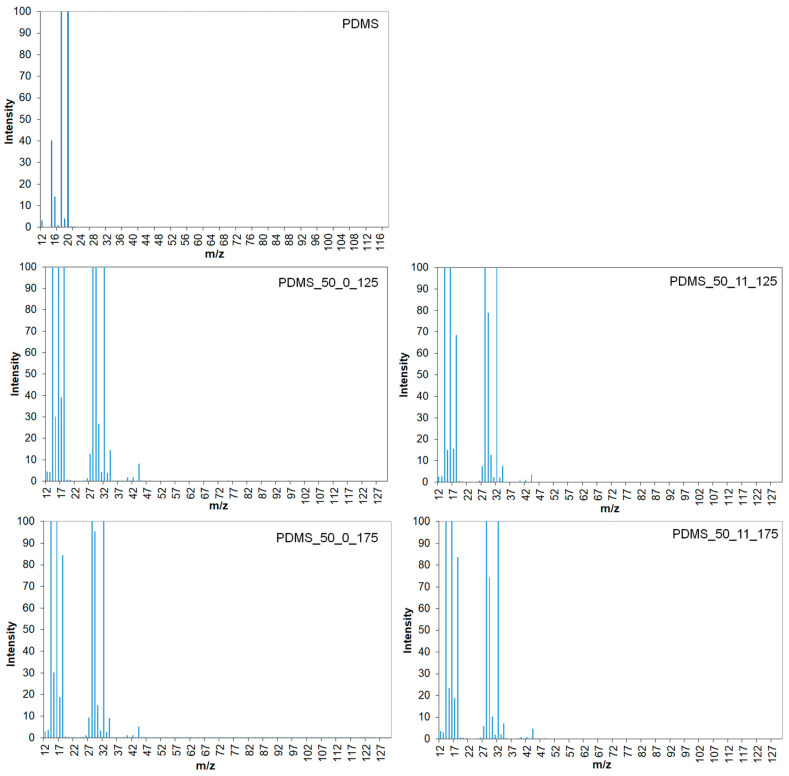
TG-MS results for fresh sample and after aging at 125 °C and 175 °C (with and without loading)—cross-section at 530 °C.

However, in our research, the PDMS did not degrade completely. For fresh PDMS, char residue was 54.3%, while for the samples after aging, it was 60.8–65.9%. A large amount of char residues can be connected to the PDMS crosslinking. It should also be noted that a higher amount of char residue was found for samples aged at higher temperatures. It suggests that PDMS can undergo secondary crosslinking and post-curing processes at elevated temperatures and under load during aging. TG-MS results and fragmentation ions detected at 530 °C are presented in Figure 5. For fresh PDMS, only fragmentation ions *m*/*z* from 12 to 22 were detected, while fragmentation ions with *m*/*z* in the range *m*/*z* from 12 to 44 were detected after aging. This can be attributed to the release of only small hydrocarbon species and water, whereas silicon-based compounds were found in the char residue. Furthermore, similar profiles of species released were found for aged PDMS, and only minor differences in intensities were observed—higher intensities were observed for samples aged at 125 °C. This suggests similar thermal stability and degradation mechanisms for PDMS after aging and indicates that higher crosslinking density hinders the thermal decomposition of PDMS.

**Figure 6:**
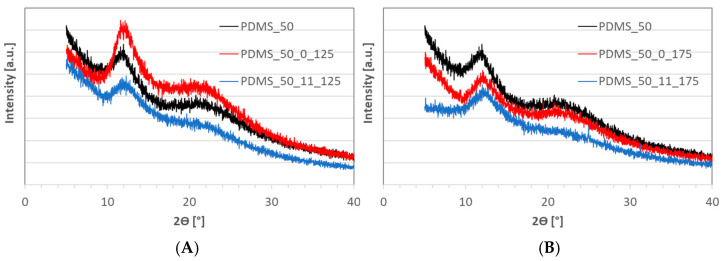
XRD diffractogram of PDMS_50 after aging in 125 °C (**A**) and 175 °C (**B**).

XRD analysis was performed for a preliminary assessment of the changes in polymer molecule arrangement after aging. Diffraction patterns for samples were presented in Figure 6. A more intense amorphous halo at around 2Ɵ = 12° was typical of the XRD profile of PDMS [29,30]. The second single halo, smaller and broader, at around 2Ɵ = 21°, was also usual for the amorphous nature of silicone, and it was probably attributable to the presence of low-molecular silicone oil. Aging PDMA at 125 °C without loading caused a slight difference in the peak intensity at 12°; it became more intensive, suggesting slight rearrangement within the chains towards a higher molecular organization. The same molecule movement was facilitated because of PDMS’s low glass transition temperature (lower than −100 °C [31]). Above this temperature, the polymer is highly elastic, and molecular changes are possible. The aging at the same temperature but underloading of the sample caused differences in the polymer structure. The increase in peak intensity at 12° was not observed, as was observed for samples aged at 125 °C without loading, suggesting an inhibition of molecule rearrangement as a result of loading. Moreover, the halo at 21° associated with the amorphous phase was significantly lower. It may indicate a smaller share of the amorphous phase due to the thermal decomposition of low-molecular silicone oil.

The diffraction pattern for sample aging at 175 °C without load was very similar to the pattern for initial PDMS. For sample aging at 175 °C with load, a decrease in the background and the disappearance of the peak at 21 associated with the amorphous phase was visible on the XRD diffractogram. It was probably due to the decomposition of silicone oil. The presented results indicate that changing the temperature from 125 °C to 175 °C without load caused slight differences in the organization of the polymer domain. Loading the samples additionally changed this process.

**Figure 7:**
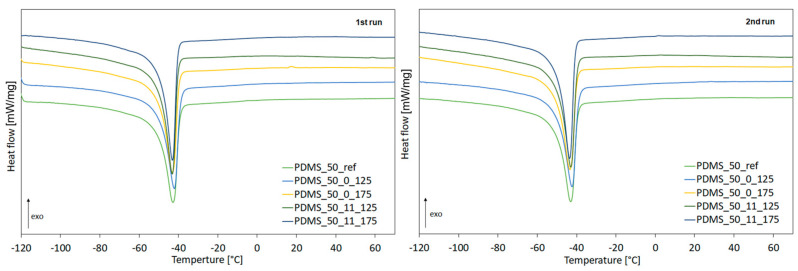
DSC curves of for fresh sample and after aging at 125 °C and 175 °C (with and without loading).

XRD results agree with the degree of crystallinity calculated from the DSC results—Figure 7 and Table 2. The highest degree of crystallinity was found in samples of fresh PDMS, both in the first and second heating runs. It can be attributed to the lower crosslinking density and the highest macromolecular mobility that enhance the formation of crystalline structures. A lower degree of crystallinity was observed for PDMS after aging; a decrease in crystallinity was observed from 55.4 to 50.1 for PDMS and PDMS_50_0_175, respectively. It indicates a process of disrupting the molecule’s organization due to its greater mobility caused by higher thermal energy. It can be attributed to the topological and geometrical constraints that hinder the crystallization of PDMS.

On the other hand, PDMS aged at higher temperatures and under load exhibited a higher degree of crystallinity compared to that for PDMS aged without load. It suggests that loading samples aged at 175 °C inhibited this process and hindered the movement of polymer molecules by reducing the distance between them. Aging has a slight effect on the melting temperature of PDMS.

**Figure 8:**
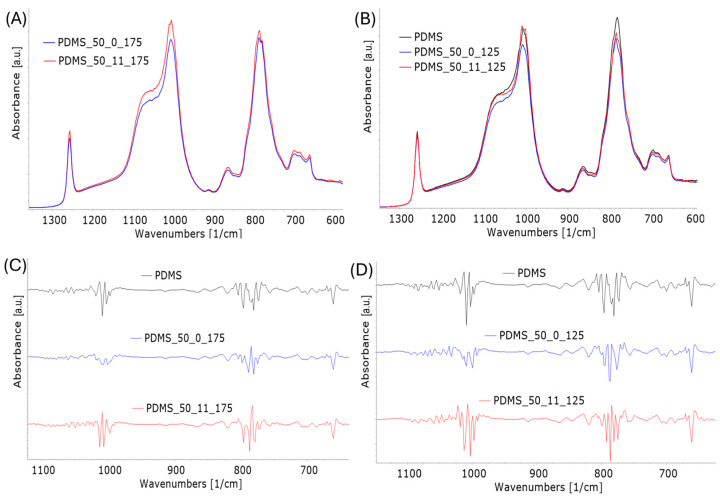
ATR-IR spectra of PDMS after aging in 175 °C (**A**) and after aging in 125 °C compared to fresh PDMS (**B**). Second derivative of ATR-IR spectra for PDMS after aging in 175 °C (**C**), and after aging in 125 °C (**D**).

ATR-IR spectra demonstrated subtle differences in the intensity of bands, explaining the changes in PDMS structure (Figure 8A,B). In particular, the intensity of bands corresponding to Si-C stretching vibrations in Si-CH_3_ at 785 cm^−1^ decreased under the aging process in comparison to the band relating to stretching vibration of Si-O-Si bonds at 1007 cm^−1^ [32,33,34]. Loading combined with high temperature caused a higher decrease in this band than high temperature alone. Moreover, a reduction in half the band’s width of 1007 cm^−1^ can be observed. These changes may suggest decomposition and crosslinking in Si-O-Si chains. It was confirmed in the second derivative of bands presented in Figure 8C,D. The mathematical transformation of PDMS_50 spectra showed that the band Si-CH_3_ with a maximum of 785 cm^−1^ comprises two main bands. Depending on the temperature of aging and loading, this region is composed of three or more bands. The second derivative also exposed the changes in the relation of components of the Si-O bands with a maximum of 1007 cm^−1^. The lack of shifts in the position of the bands suggests only changes in the shares of individual bonds. No other changes in the relations of bands were visible in ATR spectra. The other bands recorded on the spectra are typical for PDMS and are associated with asymmetric -CH_3_ stretching in Si-CH_3_ (2950–2970 cm^−1^), symmetric -CH_3_ deformation in Si-CH_3_ (1245–1270 cm^−1^), -CH_3_ rocking, and Si-C stretching vibrations in Si-CH_3_ (785–815 cm^−1^) [33].

## 4. Conclusions

The applied research methodology, including the parameters of the aging process (selected loads and temperatures) and the selection of advanced test methods for the assessment of the structure of silicone rubbers, allowed for an effective assessment of the conditions for safe use of these materials. In-depth thermal analysis was based on the determination of such crucial quantities as char residue, heat of melting, and degree of crystallinity. Thermogravimetric analysis coupled with mass spectroscopy allowed us to gain deeper insights into the thermal degradation processes. The aging of PDMS caused essential changes in its molecular structure. They were the most visible in char residue, which increased for samples after aging. The highest amount of char residue was indicated for samples aged at higher temperatures. It suggests that PDMS can undergo secondary crosslinking and post-curing processes at elevated temperatures and under load during aging. It can be concluded that the aging process hindered the thermal decomposition of PDMS due to the higher crosslinking density. Aging at higher temperatures without load caused a significant decrease in crystallinity, which can be connected with the disruption of the molecule’s organization due to its greater mobility caused by higher thermal energy. However, loading samples aged at 175 °C inhibited this process and hindered the movement of polymer molecules by reducing the distance between them. The observed changes in polymer structure did not indicate degradation. It is essential because a temperature of 175 °C is close to the critical temperature given by the producer (180 °C), above which stabilizing agents are recommended.

## Figures and Tables

**Table 1:** TG results for virgin PDMS and PDMS after aging.

Sample	T_1%_ * [°C]	T_3%_ * [°C]	T_5%_ * [°C]	T_10%_ * [°C]	T_20%_ * [°C]	T_DTGmax_ [°C]	Char Residue at 600 °C [%]
PDMS	306	360	378	430	472	373 430 481 506	54.3
PDMS_50_0_125	362	388	419	453	488	385 432 479	62.0
PDMS_50_11_125	347	378	399	446	488	386 438 478	60.8
PDMS_50_0_175	359	383	403	448	489	386 438 480	65.9
PDMS_50_11_175	353	380	397	452	484	484 452	65.9

* The values T_1%_, T_3%_, T_5%_, T_10%_, and T_20%_ represent the temperatures at which 1%, 3%, 5%, 10% and 20% mass loss occurs, respectively.

**Table 2:** Melting temperature (T_max_), heat of melting, and degree of crystallinity (**X_c_**) for virgin PDMS and PDMS after aging.

Sample	T_max_ [°C]	Heat of Melting [J/g]	X_c_ [%]
1st heating run			
PDMS_50_ref	−43	20.6	55.4
PDMS_50_0_125	−42	20.5	55.2
PDMS_50_0_175	−43	18.6	50.1
PDMS_50_11_125	−43	18.8	50.6
PDMS_50_11_175	−43	20.2	54.4
2nd heating run			
PDMS_50_ref	−43	20.5	55.2
PDMS_50_0_125	−42	19.5	52.5
PDMS_50_0_175	−43	17.9	48.2
PDMS_50_11_125	−43	19.1	51.4
PDMS_50_11_175	−44	20.2	54.4

## Data Availability

Data will be made available on request.
